# Minification of fundus optical coherence tomographic images in gas-filled eye

**DOI:** 10.1186/s12886-016-0306-1

**Published:** 2016-07-26

**Authors:** Toshifumi Yamashita, Hiroto Terasaki, Taiji Sakamoto

**Affiliations:** Department of Ophthalmology, Kagoshima University Graduate School of Medical and Dental Sciences, Kagoshima, Japan

**Keywords:** Macular hole, SD-OCT, Gas-filled eye

## Abstract

**Background:**

Optical coherence tomography (OCT) is being used increasingly to evaluate and manage a variety of retinal diseases, but not much is known about the minification of the OCT images in gas-filled eyes. The purpose of this study was to investigate the effect of gas-filled eyes on the size of the OCT images.

**Methods:**

This was retrospective case series of 81 consecutive eyes of 79 patients who had macular hole surgery between April 2012 and September 2014. Images of the optic disc were taken with a spectral domain-OCT instrument 2 days after surgery in gas-filled, pseudophakic eyes and from the same eyes but fluid-filled one month after the surgery. The vertical length, horizontal width, and the area of the optic disc were measured in the OCT images.

**Results:**

Clear images were obtained from 50 eyes of 49 patients (mean age 66.4 ± 5.9 years). The mean vertical length and mean horizontal width of the optic disc in the gas-filled eyes were about 25 % shorter than that of fluid-filled eyes (vertical, 1213.8 ± 170.5 and 1650.6 ± 195.9 μm, *P* < 0.01; horizontal, 1169.4 ± 143.1 and 1526.4 ± 219.9 μm, *P* < 0.01). The mean area of the optic disc was 1.12 ± 0.34 mm^2^ in gas-filled eyes which was significantly smaller than that in fluid-filled eyes (1.88 ± 0.37 mm^2^) by 40.4 %.

**Conclusions:**

The fundus images of gas-filled eyes are significantly smaller than that in the same fluid-filled eyes. The minification of the OCT images should be considered when analyzing images obtained from gas-filled eyes.

**Trial registration:**

Trial registration number: UMIN000007517. Date of registration: 3/21/2012.

**Electronic supplementary material:**

The online version of this article (doi:10.1186/s12886-016-0306-1) contains supplementary material, which is available to authorized users.

## Background

Recent improvements in optical coherence tomography (OCT) has made it a useful and indispensable tool for clinical ophthalmology [1–4]. OCT is used not only for diagnosis but also for monitoring the effectiveness of therapy [5, 6]. Recently, OCT instruments that allowed photographing the fundus of gas-filled eyes with a macular hole (MH) has become commercially available [7–13]. This has allowed the collection of important clinical information for the diagnosis and treatment of MHs. Clear OCT images of the macular configuration was obtained from gas-filled eyes that provided important information that could be used to determine the duration of the face-down position needed after MH surgery [9–12]. In addition, information of the closing process of a MH after surgery in the very early phase under gas provided information on this disease process [8, 11, 12].

The values of the measured parameters of the images recorded are strongly affected by the intraocular media, such as gas, oil, and saline solutions. A recent study showed that the fundus images recorded by a wide-field scanning ophthalmoscope was minified in gas-filled eyes [14].

It is essential to know the effect of the intraocular media on the OCT images before the general application of this method in clinical studies and on patients. To the best of our knowledge, there has not been a study on the minifying effect of the OCT images recorded in gas-filled eyes. Thus, the purpose of this study was to determine the effect of intraocular gas on the size of the images recorded by OCT.

## Methods

The procedures used in this study were approved by the Institutional Review Board of the Kagoshima University Hospital, and they conformed to the tenets of the 1989 Declaration of Helsinki. This study was registered with the University Hospital Medical Network (UMIN)-clinical trials registry and the registration number was UMIN000007517. A detailed explanation of the procedures was given to the patients, and a written informed consent was obtained from all. The patients also agreed to allow us to use the data for future analyses and publications.

This was a retrospective consecutive case series that included 81 consecutive eyes of 79 patients treated for a MH at the Kagoshima University Hospital between April 2012 and September 2014. The surgery consisted of standard pars plana vitrectomy with either a 23-gauge or 25-gauge system as described in detail [11, 12]. Briefly, the internal limiting membrane was peeled after core vitrectomy and a separation of the posterior hyaloid membrane. After the surgery, the contents of the vitreous cavity were exchanged with non-expansile 20 % sulfur hexafluoride (SF6). Phakic patients older than 50 years underwent cataract surgery with implantation of an intraocular lens before the vitrectomy.

### Optical coherence tomography

Images of the optic disc were taken with a spectral domain OCT instrument 2 days after surgery in gas-filled eyes and from the same eyes one month after the surgery when the gas had been absorbed and the cavity was fluid-filled. We analyzed only the high-quality OCT images of gas-filled eye as reported elsewhere [11, 12]. During the follow-up period, we recorded images not only of the macular area but also of the optic disc with the Cirrus HD-OCT (Carl Zeiss Meditec, Dublin, Calif., USA). For this, the focus was adjusted to –20 diopters and scanning was performed. The focus on the fundus and B scan images was always adjusted simultaneously to get the best focused image. A detailed description of the methods was reported in our previous publications [11, 12]. From our experience, a Cirrus HD-OCT is the best instrument to obtain clear images of gas-filled eyes compared to other OCT instruments because of the differences in the range of focus, recordings can be made with or without the auto-focus function, and ability to alter the spacing of each slice in the B-scan recordings.

The optic disc area was scanned with the optic disc cube 200 × 200 protocol. All SD-OCT examinations were performed with the patients sitting in an upright position. Most OCT instruments including the Cirrus HD-OCT have an embedded function to assess the image intensity and quality, which is expressed as signal strength in the Cirrus HD-OCT. Lower signal-to-noise ratios indicate poor image quality, and these images should not be used to assess the retina especially in older subjects. So, images with a signal strength <5 were excluded.

### Measurements of vertical lengths and horizontal widths and areas of optic disc

**Fig. 1:**
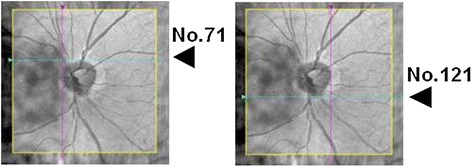
Measurement of the vertical length and horizontal width of the optic disc. The size of optic disc was measured in the Optic Disc Cube (200 x 200) images of the Cirrus HD-OCT. Because the spacing of each slice is 30 μm, the length of the optic disc was calculated as: Length of optic disc = Number of scan X 30μm. Because the number of scans is 50 (i.e.,121-71) in this case, the vertical length of optic disc is 1500 μm

The vertical lengths and horizontal widths of the optic disc were measured semi-automatically with a program built-into the Cirrus OCT. In the optic disc cube 200 × 200 protocol, a 6 × 6 mm area was recorded with 200 consecutive scans. Then, the size of each slice is 30 μm (6000 μm/200 scans = 30 μm). The length of the optic disc in the images was calculated as (Fig. 1):

$$ \mathrm{length}\ \mathrm{of}\ \mathrm{optic}\ \mathrm{disc} = \mathrm{Number}\ \mathrm{of}\ \mathrm{scan}\ \mathrm{X}\ 30\upmu \mathrm{m}. $$

The optic disc area was calculated by a software embedded in the OCT instrument.

### Statistical analyses

All statistical analyses were performed with the SPSS program for Windows (SPSS Inc., IBM, Somers, New York, USA). Wilcoxon signed-rank tests were used to determine the significance of differences in the data. A *P* value <0.05 was taken to be statistically significant.

## Results

Eighty-one consecutive eyes of 79 patients that had undergone vitrectomy for a MH were studied. Thirty-one eyes of 30 patients were excluded because the quality of the images was <5. Thus, 50 eyes of 49 cases were studied of which 27 were women and 22 were men. Their mean ± SD age was 66.4 ± 5.9 years with a range of 56 to 85 years. Forty-seven eyes were phakic preoperatively and 3 eyes were pseudophakic. The 47 eyes underwent phacoemulsification and lens implantation before the vitrectomy, and the 3 pseudophakic eyes had vitrectomy alone. As a result, all OCT images taken were from pseudophakic eyes.

### Vertical length, horizontal width, and area of optic disc

**Fig. 2:**
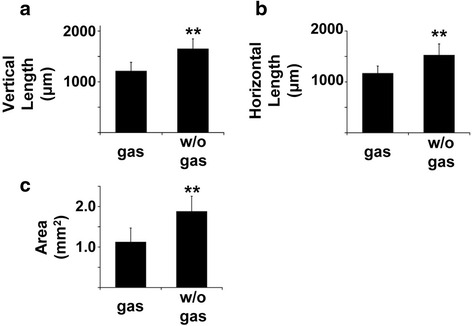
Degree of reduction of OCT images in gas-filled eyes. The vertical length of the optic disc was 23.5 % shorter in gas-filled eyes than the same eyes after the gas had been absorbed and the eye was fluid-filled (**a**, *P* < 0.01). The horizontal width was 26.5 % narrower in gas-filled eyes than after the gas is absorbed and the eye is fluid-filled (**b**, *P* < 0.01). The area of the optic disc of gas-filled eye was significantly smaller than eyes after the gas was absorbed by 40.4 % (**c**, *P* < 0.01)

The mean vertical length of the optic disc was 1213.8 ± 170.5 μm two days after surgery when the eye was gas-filled and 1650.6 ± 195.9 μm at one month after the surgery when the eye was fluid-filled. The mean horizontal width of the optic disc was 1169.4 ± 143.1 μm 2 days after surgery when the eye was gas-filled and 1526.4 ± 219.9 μm one month after the surgery when the eye was fluid-filled. Thus, the vertical length was shorter by 26.5 % and the horizontal width by 23.5 %. The vertical length and horizontal width of the optic disc in the gas-filled eye were significantly shorter than that in the fluid-filled eyes (*P* < 0.01, Fig. 2a–b).

**Fig. 3:**
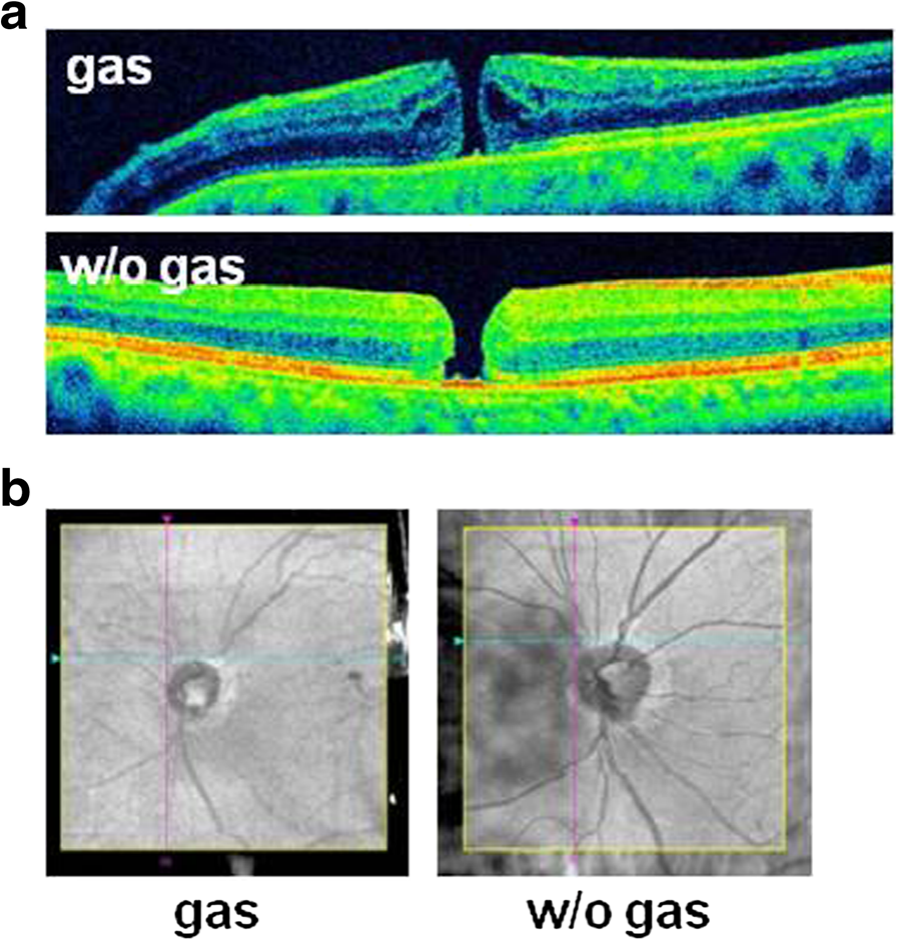
Representative images of a gas-filled eye and a fluid-filled eye. Images of an unclosed MH in a vertical scan image. The hole diameter in the gas-filled eye is 120 μm which is significantly smaller than that in the fluid-filled eye at 192 μm 1 month after surgery (**a**). The area of optic disc in the gas-filled eye is 1.18 μm^2^ which is significantly smaller than that in fluid-filled eyes at 1.64 μm^2^) (**b**)

The mean area of the optic disc was 1.12 ± 0.34 mm^2^ in the gas-filled eye which was significantly smaller than that in the fluid-filled eyes at 1.88 ± 0.37 mm^2^ (*P* < 0.01). The reduction was by 40.4 % (Fig. 2c). Representative images including one with an unclosed MH and with the optic disc are shown in Fig. 3 and Additional file 1.

## Discussion

Our results showed that the images from gas-filled eyes were about 25 % smaller in both the vertical and horizontal directions and about 40 % smaller in the area than the images obtained from the same eyes after the gas was absorbed and the vitreous cavity was fluid-filled. Images of gas-filled eyes by the ultra-wide–field scanning ophthalmoscope Optos 200Tx (Optos, Marlborough, MA, USA) were also reported to be minified [14]. Meyer et al reported that the images obtained with a panfunduscopic lens were 30 % smaller in gas-filled eye than in eyes without gas or air [15]. They did not present detailed methods for the measurements and calculations. However, the degree of reduction is comparable to our findings. Since the measurements in our patients were made by a built-in program of the Cirrus HD-OCT, this method was more objective.

**Fig. 4:**
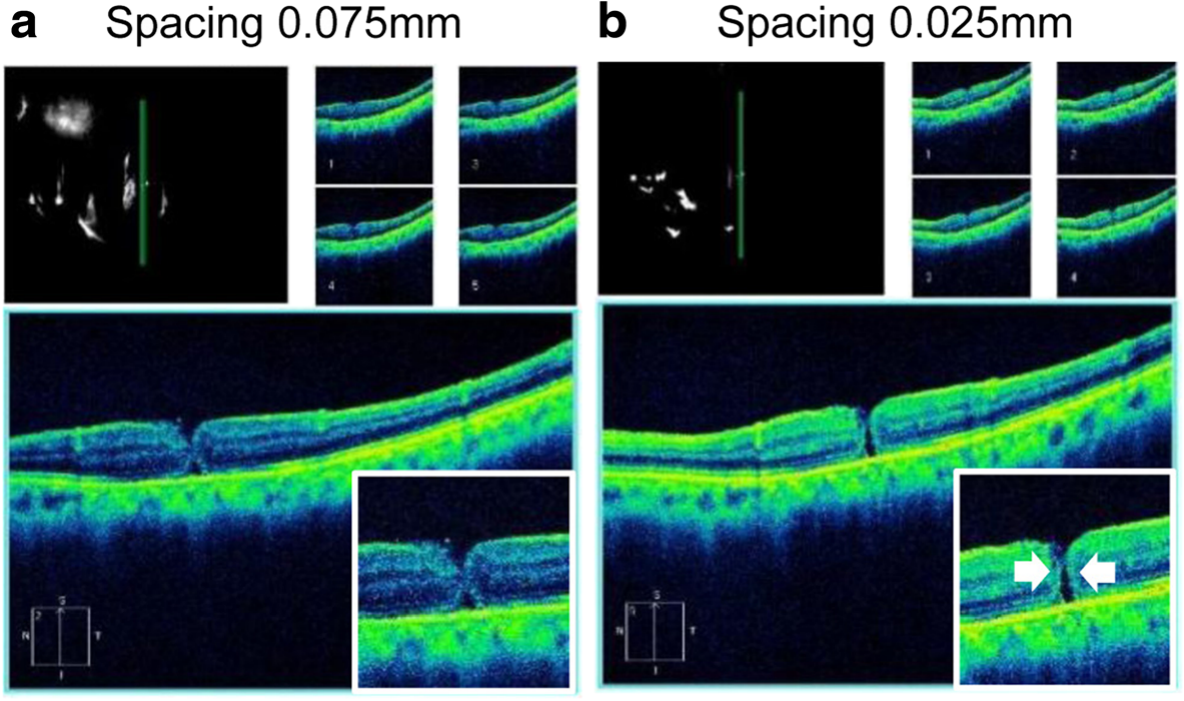
Appearance of MH with scan spacing of 0.075 mm and 0.025 mm 2 days after vitrectomy. The MH appears approximately closed in images of 0.075 mm space scan (**a**) but clearly not closed in 0.025 mm space scan (**b**, *white arrows*)

The reduction in the image size can lead to an error in the size of an unclosed MH in gas-filled eye. For example, the image of the MH in the gas-filled eye was smaller than that in the same eye without gas (Fig. 3a). The actual length of the optic disc in the OCT images of gas-filled eyes would be expected to be larger when the gas is replaced by fluid. The measurements can be corrected by multiplying the values by 1.3 because the length of optic disc in gas-filled eyes was about 25 % smaller. A MH closure might occasionally be overlooked in gas-filled eye in the original width of each scan of 0.25 mm in the 5-line raster scan. A width of 0.025 mm, which is narrowest setting in this protocol, should be suitable for confirming a closure of a MH. This can also occur with a 0.075 mm spacing (Fig. 4).

There are several reports studying the OCT images in silicone oil-filled eyes showing the very early processes of MH closure after vitrectomy [16–18]. The results indicated that the disappearance of the fluid-cuff and peri-MH cysts are important signs of MH closure. However, a detailed description of the changes that occur during MH closure in gas-filled eyes had not been published which is important to determine the choice of the most effective treatment. Kukushima et al recently reported on the very early stage of MH closure soon after vitrectomy with SF6 gas tamponade using OCT imaging in the same way we did [8]. They reported the MH closure occured in 3 distinct steps. In step 1, the torn and separated edges of the photoreceptor layer which forms the minimum MH diameter preoperatively, formed a kissing configuration from as early as 20 min after the vitrectomy up until day 1. In step 2, the residual MH above the connected photoreceptor layer closes. Finally in step 3, the subretinal fluid below the connected photoreceptor layer is absorbed and the MH is completely closed [8]. The minification of the OCT images in gas-filled eyes should be noted in investigations of OCT images of gas-filled eyes or comparison of dynamics of MH closure between silicon oil tamponade and SF6 tamponade.

There are limitations in this study. First, this was a retrospective study and potential sampling bias cannot be eliminated. Second, the eyes were limited to those with a MH treated with 20 % SF6 gas. Third, taking high-quality images of optic disc in gas-filled eyes were relatively difficult compared to that of B-scan as reported [11, 12] probably because of the reflection or non-specific noises. Fourth, although this study excluded highly myopic eyes [11, 12], ocular magnification might affect our results and the minification effect in gas-filled eye on the retinal thickness were not determined [19].

## Conclusions

The optic discs in SD-OCT images of gas-filled eye are about 25 % shorter in the vertical and horizontal lengths, and 40 % smaller in area than the size of the same eyes after the gas is absorbed. This information is essential for correct evaluation of the status of the fundus images in gas-filled eyes.

## Abbreviations

MH, macular hole; OCT, optical coherence tomography; SF6, sulfur hexafluoride

## Additional file

Additional file 1: Representative images of optic disc in a gas-filled eye and a fluid-filled eye. (JPG 154 kb)
