# Supplementary figures and images for: Minification of fundus optical coherence tomographic images in gas-filled eye
Source: BMC Ophthalmol. 2016 Jul 26;16:124. doi: 10.1186/s12886-016-0306-1 (PMC4960867; doi:10.1186/s12886-016-0306-1)

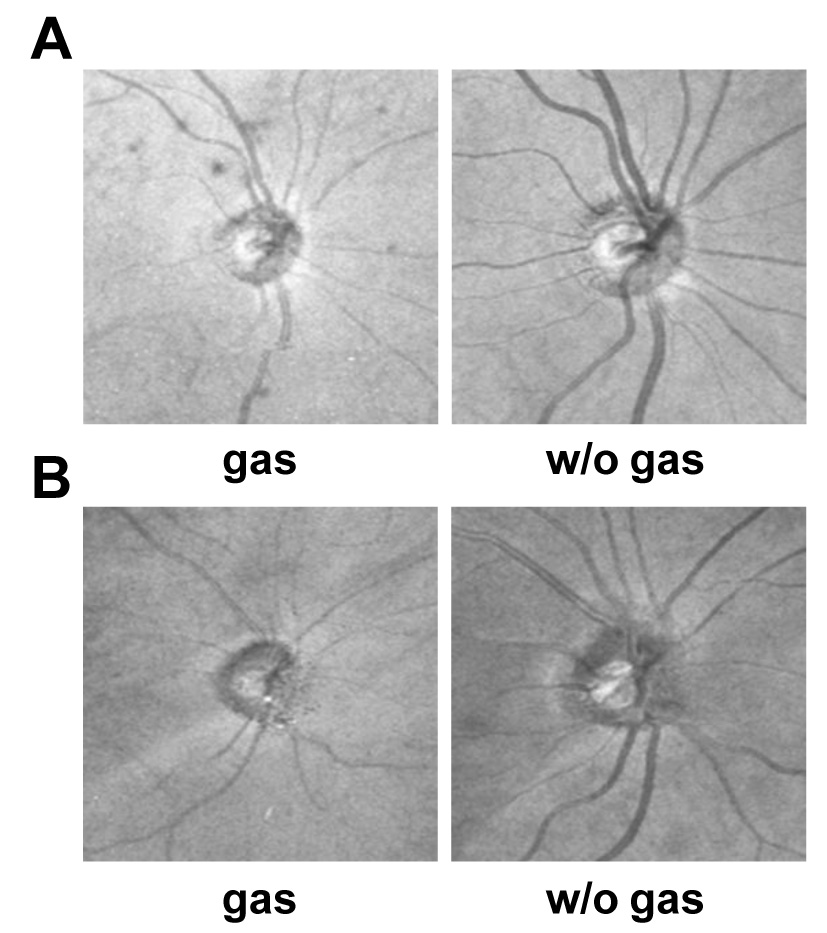

Supplement: Additional file 1: — Representative images of optic disc in a gas-filled eye and a fluid-filled eye. (JPG 154 kb) [file 12886_2016_306_MOESM1_ESM.jpg]
